# Cognitive Radio Wireless Sensor Networks: Applications, Challenges and Research Trends

**DOI:** 10.3390/s130911196

**Published:** 2013-08-22

**Authors:** Gyanendra Prasad Joshi, Seung Yeob Nam, Sung Won Kim

**Affiliations:** Department of Information and Communication Engineering, Yeungnam University, 214-1 Dae-dong, Gyeongsan-si, Kyongsan 712-749, Gyeongsangbuk-do, Korea; E-Mails: joshi@ynu.ac.kr (G.P.J.); synam@ynu.ac.kr (S.N.)

**Keywords:** sensor networks, cognitive sensors, cognitive wireless sensor networks

## Abstract

A cognitive radio wireless sensor network is one of the candidate areas where cognitive techniques can be used for opportunistic spectrum access. Research in this area is still in its infancy, but it is progressing rapidly. The aim of this study is to classify the existing literature of this fast emerging application area of cognitive radio wireless sensor networks, highlight the key research that has already been undertaken, and indicate open problems. This paper describes the advantages of cognitive radio wireless sensor networks, the difference between *ad hoc* cognitive radio networks, wireless sensor networks, and cognitive radio wireless sensor networks, potential application areas of cognitive radio wireless sensor networks, challenges and research trend in cognitive radio wireless sensor networks. The sensing schemes suited for cognitive radio wireless sensor networks scenarios are discussed with an emphasis on cooperation and spectrum access methods that ensure the availability of the required QoS. Finally, this paper lists several open research challenges aimed at drawing the attention of the readers toward the important issues that need to be addressed before the vision of completely autonomous cognitive radio wireless sensor networks can be realized.

## 1. Introduction

### 1.1. Conventional Wireless Sensor Networks

Communications in wireless sensor networks (WSNs) are event driven. Whenever an event triggers wireless sensor (WS) nodes generate bursty traffic. In a dense network environment, wireless sensor nodes deployed in the same area might try to access a channel whenever an event occurs. Recently, many sensitive and critical activities are being monitored and observed increasingly using WSNs. Several heterogeneous WSNs can exist, which causes a long waiting time for the delay sensitive data. Wireless sensors are normally deployed in inaccessible terrain. Therefore, the self-organizing ability and lifetime of the WS nodes are very important.

**Figure 1.**
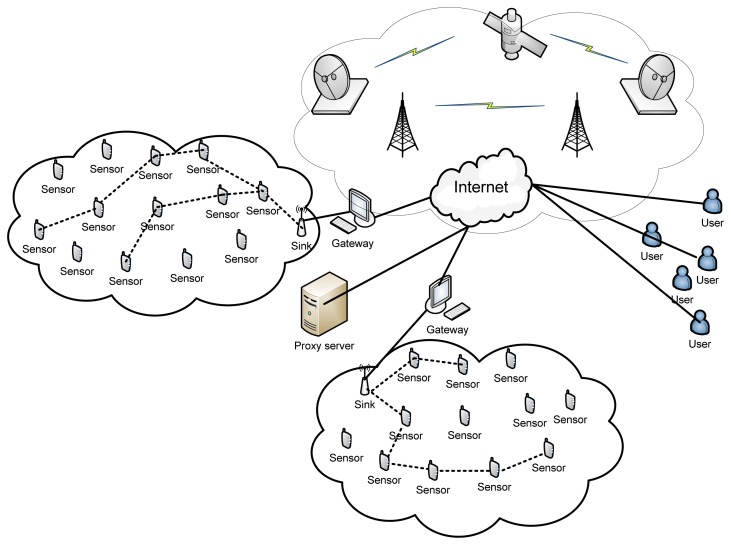
Conventional wireless sensor networks.

WSNs consist of hundreds of WS nodes deployed throughout the sensor field and the distance between two neighboring WS nodes is generally limited to few meters. A sink node or base station is responsible for collecting the data from the WS nodes in single or multiple-hop manner. The sink node then sends the collected data to the users via a gateway, often using the internet or any other communication channel. Figure 1 shows the scenario of conventional WSNs.

Current WSNs operate in the ISM band, which is shared by many other successful communication technologies. Research has shown that this coexistence in the ISM band can degrade the performance of the WSNs. The wide deployments, large transmit power, and large coverage range of IEEE 802.11 devices and other proprietary devices can degrade the performance of WSNs significantly when operating in overlapping frequency bands. The coexistence of wireless personal area networks (WPAN) with other wireless devices operating in an unlicensed frequency band is addressed in reference [1].

WSN devices are not only a victim but are also an interferer sometimes [2]. The coexistence interference can be avoided by the intelligent use of three types of diversity, namely frequency, time and space. Coexistence issues in unlicensed bands have been the subject of extensive research. Some solutions are also suggested in references [3–5].

Researchers and industry are working to improve the performance of WSNs in terms of cost, energy consumption, data rate, robustness, networks throughput, QoS and security, *etc.* Considerable hardware and software enhancement has been implemented in recent years to enhance the network performance. A range of logical techniques have been employed to achieve the required network performance, such as power aware MAC, cross-layer design technique, efficient sensing technique, and significant enhancement in hardware design, *etc.*, but these techniques have their own limitations.

### 1.2. Adopting Cognitive Method in WSN

Recently, cognitive techniques have been used in wireless networks to circumvent the limitations imposed by conventional WSNs. Cognitive radio (CR) is a candidate for the next generation of wireless communications system. The cognitive technique is the process of knowing through perception, planning, reasoning, acting, and continuously updating and upgrading with a history of learning. If cognitive radio can be integrated with wireless sensors, it can overcome the many challenges in current WSNs. CR has the ability to know the unutilized spectrum in a license and unlicensed spectrum band, and utilize the unused spectrum opportunistically. The incumbents or primary users (PU) have the right to use the spectrum anytime, whereas secondary users (SU) can utilize the spectrum only when the PU is not using it.

Some recent papers in this paradigm, such as references [6–11], proposed wireless sensor equipped with cognitive radio as one of the promising candidates for improving the efficiency of WSNs. Table 1 lists the capabilities a wireless sensor with a CR needs to have.

CR allows unlicensed users to access multiple licensed channels opportunistically. This nature of CR gives potential advantages to WSNs by increasing the communication reliability and improving the energy efficiency. When wireless sensor nodes with cognitive capabilities are introduced to an entire network, it gives exciting new opportunities to researchers and industry to develop algorithms, hardware and software that can overcome the limitations imposed by current wireless sensor design techniques.

Taking advantage of the current liberalization in the spectrum utilization rule by FCC and technical advancement in sensor technology, wireless sensors with CR can mitigate the current issue of spectrum inefficiency and increase the network efficiency in a range of terms.

### 1.3. New Paradigm of WSN with CR: Cognitive Radio Wireless Sensor Networks (CR-WSN)

CR-wireless sensor networks (CR-WSNs) are a specialized *ad hoc* network of distributed wireless sensors that are equipped with cognitive radio capabilities. CR-WSN is different in many aspects with a conventional WSN and conventional distributed cognitive radio networks (CRNs). The following section details the differences in the aspects among *ad hoc* CRNs, WSNs, and CR-WSNs. CR-WSNs normally involve a large number of spatially distributed energy-constrained, self-configuring, self-aware WS nodes with cognitive capabilities. They require cognition capacity for a high degree of cooperation and adaptation to perform the desired coordinated tasks. They have not only to transfer data packets, but also to protect incumbent license users. More explicitly, this is a system that employs most of the capabilities required for a CR system, as defined by International Telecommunication Union (ITU) [12] and also for WSNs.

According to Akan *et al.* [7], a CR-WSN is defined as a distributed network of wireless cognitive radio wireless sensor (CRWS) nodes, which sense an event signal and collaboratively communicate their readings dynamically over the available spectrum bands in a multi-hop manner, ultimately to satisfy the application-specific requirements.

**Figure 2.**
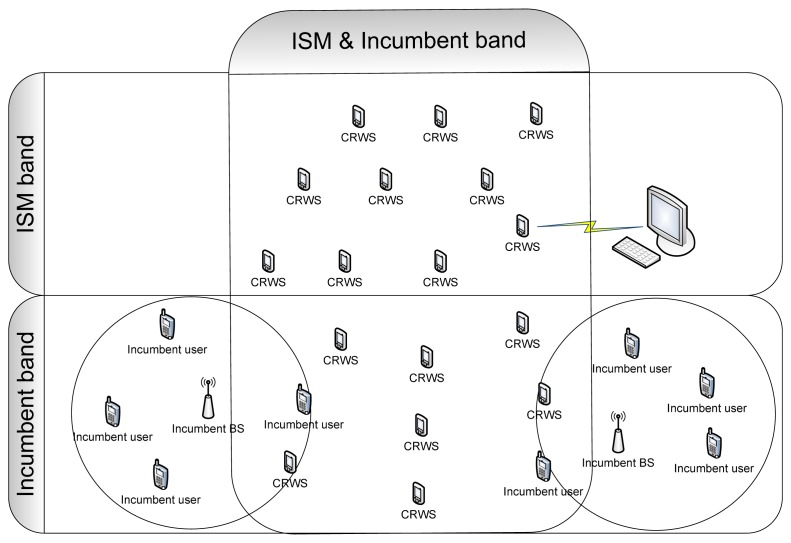
CR-WSNs model.

In CR-WSNs, a wireless sensor node selects the most appropriate channel once an idle channel is identified and vacates the channel when the arrival of a licensed user on the channel is detected. The cognitive radio technique is probably one of the most promising techniques for improving the efficiency of the WSNs. CR-WSNs increase spectrum utilization, and fulfills the end-to-end goal, increase network efficiency and extend the lifetime of WSNs. Figure 2 presents a CR-WSNs model.

### 1.4. Advantages of Using CR in WSNs

CR-WSN is a new paradigm in a WS network arena that utilizes the spectrum resource efficiently for bursty traffic. The system has the capability of packet loss reduction, power waste reduction, high degree of buffer management, and has better communication quality. This section discusses the advantages of using cognitive radio in WSNs.

#### 1.4.1. Efficient Spectrum Utilization and Spaces for New Technologies

The electromagnetic spectrum is a precious gift of Nature. The amount of available useable spectrum bands cannot be increased but they can be used more efficiently. With the exception of industrial, scientific and medical (ISM) radio bands, one requires a license from the government of the respective country to utilize the radio bands. Owing to the high cost associated with spectrum licensing, many researchers and hardware manufacturers have focused on developing devices for ISM bands. Therefore, ISM bands are overcrowded limiting the development of new technologies [13]. On the other hand, many licensed spectrum bands are either underutilized or unutilized [14]. Cognitive radio wireless sensors can use the unutilized spectrum, called white spaces, without disturbing the license holders. Unlicensed users can use those bands with little or no cost, so that more technologies can be developed for these bands. Table 2 lists the frequency bands available for ISM applications, as defined by ITU-R (RR Nos. 5.138 and 5.150) [15].

#### 1.4.2. Multiple Channels Utilization

Most traditional WSNs use a single channel for communication [16]. In WSNs, upon the detection of an event, sensor nodes generate the traffic of packet bursts. At the same time, in densely deployed WSNs, a large number of wireless sensor nodes within the event area attempt to acquire the same channel at the same time. This increases the probability of collisions, and decreases the overall communication reliability due to packet losses, leading to excessive power consumption and packet delay. CR-WSNs access multiple channels opportunistically to alleviate this potential challenge.

#### 1.4.3. Energy Efficiency

In WSNs, there is a large amount of power waste for packet retransmission due to packet losses. CR wireless sensors may be able to change their operating parameters to adapt to channel conditions. Therefore, energy consumption due to a packet collision and retransmission can be mitigated.

#### 1.4.4. Global Operability

Each country has its own spectrum regulation rules. A certain band available in one country might not be available in another. Traditional wireless sensors with a preset working frequency might not work in cases where the manufactured wireless sensors are deployed in different regions. On the other hand, if nodes are equipped with cognitive radio capability, they can overcome the spectrum incompatibility problem by changing their communication frequency band. Therefore, CR wireless sensors have the potential to be operated almost anywhere in the world.

#### 1.4.5. Application Specific Spectrum Band Utilization

Currently, the number of wireless sensors deployed for different applications has increased. In WSN, data traffic is usually correlated both temporally and spatially. When any event occurs, WSNs generate packet bursts and they remain silent when there is no event. These temporal and spatial correlations introduce to the design challenge of the communication protocols for WSN. With the intelligent communication protocols in CR-WSN, it is possible that the wireless sensors deployed for the same purpose use the spectrum of different incumbents in spatially overlapping regions. This is possible with cooperative communication among SUs, which obviously mitigates interference issues.

#### 1.4.6. Financial Advantages to the Incumbents by Renting or Leasing

Whenever and wherever some licensed spectrum bands are not required, license holders can lease their spectrum to the SUs at low cost. This can be done while retaining the access of the incumbents on the spectrum bands whenever necessary. This is very good opportunity for those who cannot obtain a direct license for a certain spectrum due to legal or financial issues. This is a win-win approach to the incumbents and SUs.

#### 1.4.7. Avoiding Attacks

Unlike CRWS, most off-the-shelf wireless sensors work only on particular frequency bands. Taking advantage of the wide range of spectrum usability, SUs in CR-WSNs can avoid several types of attacks. Attacks in CR-WSNs are discussed in Section 3.11.

### 1.5. Differences between Ad Hoc CRNs, WSNs and CR-WSNs

This section examines the properties, differences and commonalities of *Ad Hoc* CRNs, WSNs, and CR-WSNs. Although some of the channel sensing, channel decision, channel access, spectrum management, reliability, network security, and issues in CR-WSNs are similar to the issues in *ad hoc* CRNs or conventional WSNs, there are some differences in a number of factors. Some issues in CRNs have been addressed well [18–33]. Table 3 compares several factors among *ad hoc* CRNs, conventional WSNs and CR-WSNs.

## 2. Potential Application Areas of CR-WSNs

CR-WSNs may have a wide range of application domains. Indeed, CR-WSN can be deployed anywhere in place of WSNs. Some examples of prospective areas where CR-WSNs can be deployed are as follows: facility management, machine surveillance and preventive maintenance, precision agriculture, medicine and health, logistics, object tracking, telemetries, intelligent roadside, security, actuation and maintenance of complex systems, monitoring of indoor and outdoor environments. This section discusses some of the potential areas where CR-WSNs can be deployed with examples.

### 2.1. Military and Public Security Applications

Conventional WSNs are used in many military and public security applications, such as: (a) chemical biological radiological and nuclear (CBRN) attack detection and investigation; (b) command control; (c) gather the information of battle damage evaluation; (d) battlefield surveillance; (e) intelligence assistant (f) targeting, *etc.* In the battlefield or in disputed regions, an adversary may send jamming signals to disturb radio communication channels [34,35]. In such situations, because CR-WSs can handoff frequencies over a wide range, CR-WSNs can use different frequency bands, thereby avoiding the frequency band with a jamming signal. In addition, some military applications require a large bandwidth, minimum channel access and communication delays. For such applications, CR-WSNs can be a better option.

### 2.2. Health Care

In a health care system, such as telemedicine, wearable body sensors are being used increasingly. Numerous wireless sensor nodes are placed on patients and acquire critical data for remote monitoring by health care providers. In 2011, the IEEE 802.15 Task Group 6 (BAN) [36] approved a draft of a standard for body area network (BAN) technology. Wireless BAN-assisted health care systems have already been in practice in some remote areas of developing countries, such as in Nepal and India [37,38]. Wireless BAN for healthcare systems is suitable for areas, where the number of health specialists is relatively low.

**Figure 3.**
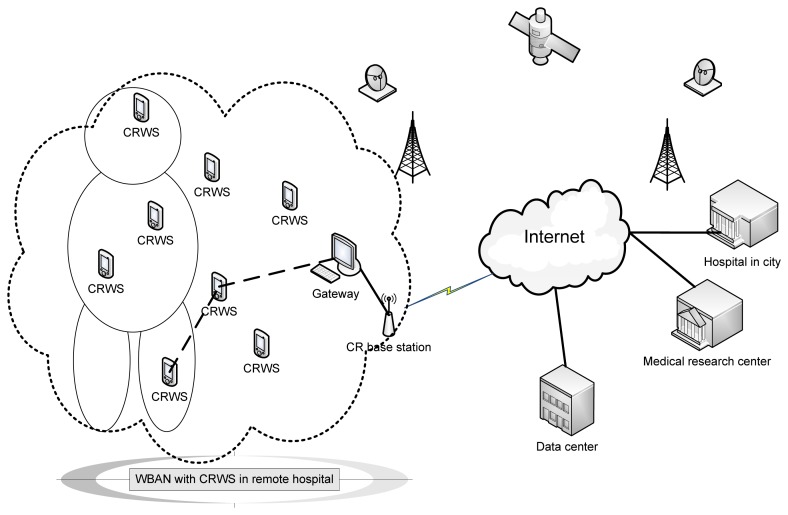
Wireless body area network with CRWS.

Medical data is critical, delay and error sensitive. Therefore, the limitation of traditional WSN, as discussed in the previous section confines the potentiality of telemedicine. The QoS may not be achieved at a satisfactory level if the operating spectrum band is crowded in convenient ‘telemedicine with BAN’. The use of ‘CR wearable body wireless sensors’ can mitigate these problems due to bandwidth, jamming and global operability, hence improve reliability. Figure 3 presents a model for wireless BAN with CR wireless sensors. A significant amount of research has been carried out in the area of WBASN [39]. The requirements of cognitive radio implementation in wireless medical networks are discussed in reference [40].

### 2.3. Home Appliances and Indoor Applications

Many potential and emerging indoor applications require a dense WSNs environment to achieve an adequate QoS. Conventional WSNs experience significant challenges in achieving reliable communication because ISM bands in indoor areas are extremely crowded [13]. Some examples of the indoor applications of WSNs are intelligent buildings, home monitoring systems, factory automation, personal entertainment, *etc.* CR-WSNs can mitigate the challenges faced by conventional indoor WSNs applications.

### 2.4. Bandwidth-Intensive Applications

Multimedia applications, such as on-demand or live video streaming, audio, and still images over resource constrained WSNs, are extremely challenging because of their huge bandwidth requirements [41–43]. Other WSN applications, such as WSNs in a hospital environment, vehicular WSNs, tracking, surveillance, *etc.*, have vast spatial and temporal variations in data density correlated with the node density. These applications are bandwidth-hungry, delay intolerable and bursty in nature. Because in CR-WSN, SUs can access multiple channels whenever available and necessary, CR-WSN is very suitable for these types of bandwidth-hungry applications. Rehmani *et al.* [44] reported channel bonding in CR-WSNs for such bandwidth intensive applications.

### 2.5. Real-Time Surveillance Applications

Real-time surveillance applications, such as traffic monitoring, biodiversity mapping, habitat monitoring, environmental monitoring, environmental conditions monitoring that affect crops and livestock, irrigation, underwater WSNs, vehicle tracking, inventory tracking, disaster relief operations, bridges or tunnel monitoring, require minimum channel access and communication delay. Some real-time surveillance applications are highly delay-sensitive and require high reliability. A delay due to a link failure can also occur in multihop WSNs if the channel condition is not good. On the other hand, WS nodes hop to another channel if they find another idle channel with a better condition in CR-WSNs. Channel aggregation and the use of multiple channels concurrently are possible in CR-WSNs to increase the channel bandwidth [45,46].

### 2.6. Transportation and Vehicular Networks

The IEEE 1609.4 standard proposes multi-channel operations in wireless access for vehicular environments (WAVE). The WAVE system operates on the 75 MHz spectrum in the 5.9 GHz band with one control channel and six service channels. All vehicular users will have to contend for channel access and use it to transmit the information in the 5.9 GHz band. However, it still suffers from spectrum insufficiency problems. This spectrum scarcity issue and the requirements of cognitive radio in WAVE have been studied [47–49].

Some preliminary works in CR-enabled vehicular communications have already been done [48]. Vehicular wireless sensor networks are emerging as a new network paradigm for proactively gathering monitoring information in urban environments. CR-WSNs are likely to be more relevant in this field. Although this area still needs to be examined, some protocols for highway safety using CR-WSNs have been proposed [50,51].

### 2.7. Diverse Purpose Sensing

Increasingly, the use of wireless sensors in the same area for different objectives coexists. In a conventional WSN, those wireless sensors attempt to access the channel in non-cooperative manners. With the help of an efficient medium access control (MAC) protocol, CR-WSN might select different channels for different applications considering load balancing and fairness.

## 3. Challenges

CR-WSNs differ from conventional WSNs in many aspects. Because protecting the right of PUs is the main concern in CR-WSN, it has many new challenges including the challenges in the conventional WSNs. This section discusses the challenges affecting the design of a CR-WSN.

### 3.1. Detection, False Alarm, and Miss-Detection Probability

The detection probability is a metric used for correct detection by CRWS regarding the absence of PUs on the channel. The miss-detection probability is a metric for CRWS failing to detect the presence of the primary signal on the channel, and the false-alarm probability is a metric for the CRWS failing to detect the absence of the primary signal.

Sensing can be viewed as a binary hypothesis testing problem with hypotheses *H_0_* and *H_1_*:

(1) $$\{\begin{array}{c} H_{1}:\text{Currently occupied by PU}\\ H_{0}:\text{Available for SUs} \end{array}$$

Letaief *et al.* [52] defined the miss-detection probability (*P_m_*) and false alarm probability (*P_f_*) in CR networks as follows:

(2) $$P_{m}=P(H_{0}|H_{1})$$

(3) $$P_{f}=P(H_{1}|H_{0})$$

In CR-WSNs, a false alarm and miss detection can violate the right of the incumbents on the channel, which is the violation of the main principle of CRNs. The right to access a network by the incumbents should be respected in any type of CRN. A false alarm can cause spectrum under-utilization and a missed detection might cause interference with the PUs. In addition, most application areas of CRWS discussed in this paper are very critical in terms of the delay and correctness of data. In CR-WSNs, a false alarm and miss-detection causes a long waiting delay, frequent channel switching and significant degradation in throughput. The issues of the false alarm and miss-detection probability for CR *ad hoc* networks and IEEE 802.22 WRAN have been well studied. However, this area still needs to be explored for CR-WSNs. This area needs to be examined further to meet the research challenges of CR-WSNs.

### 3.2. Hardware

CR wireless sensors have hardware constraints in terms of computational power, storage and energy. Unlike conventional wireless sensors, they have a responsibility to sense channels, analyze, decide, and act. CR wireless sensors should be capable of changing the parameter or transmitters based on an interaction with its environment.

**Figure 4.**
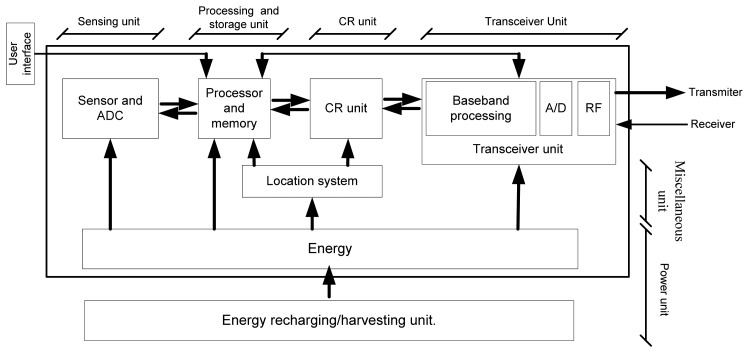
Hardware structure of CR wireless sensor.

As shown in Figure 4, a CRWS consists of six basic units: (i) a sensing unit; (ii) a processing and storage unit; (iii) a CR unit; (iv) a transceiver unit; (v) a power unit; and (vi) a miscellaneous unit.

Sensing units contain sensors and analog to digital converters (ADCs). The analog signal observed by the sensor is converted to a digital signal and sent to the processing unit. The CRWS should have cognition capability using a state-of-the art artificial intelligence technology. This capability is accommodated in the CR unit. The CR unit needs to adapt the communication parameters dynamically, such as carrier frequency, transmission power, and modulation. The unit needs to select the best available channel, share the spectrum with other users, and manage the spectrum mobility, i.e., vacate the currently using channel in the case the PU wants to use that channel. A transceiver unit is responsible for receiving and sending data.

Because the energy harvesting techniques in wireless sensor nodes have developed rapidly, the energy harvesting or recharging units are optional and sensor specific. A miscellaneous unit is an application- specific additional unit, such as a location-finding unit, energy harvesting unit, and mobilizing unit, *etc.* Akan *et al.* [7] proposed a similar hardware structure of a CR wireless sensor.

Designing intelligent hardware for CR-WSNs is a very challenging issue. Many artificial intelligence techniques have been proposed to fulfill the basic principle of CR, i.e., observation, reconfiguration and cognition. Some examples include artificial neural networks (ANNs), metaheuristic algorithms, hidden Markov models (HMMs), rule-based systems, ontology-based systems (OBSs), and case-based systems (CBSs). The factors that affect the choice of AI techniques, such as responsiveness, complexity, security, robustness, and stability, are discussed in reference [32]. Nevertheless, it is unclear how much intelligent hardware for WS-CRNs is intelligent enough and no threshold has been defined for it.

### 3.3. Topology Changes

Topology directly affects the network lifetime in WSNs. Depending on the application, CR wireless sensors may be deployed statically or dynamically. In any type of WSN, hardware failure is common due to hardware malfunctioning and energy depletion. The topologies for CR-WSNs may be the same as conventional WSNs, but they are prone to change more frequently than *ad hoc* CRNs. Akan *et al.* [7] reported that CR-WSNs have the following topologies: (i) *Ad Hoc* CR-WSNs; (ii) Clustered CR-WSNs; (iii) Heterogeneous and Hierarchical CR-WSNs; and (iv) Mobile CR-WSNs.

Basically, the minimum output power required to transmit a signal over a distance *δ* is proportional to *δ^n^*, where *2* ≤ *n* < *4*. The exponent *n* is closer to four for low-lying antennae and near-ground channels, as is typical in wireless sensor network communication. Therefore, routes that have more hops with shorter hop distances can be more power efficient than those with fewer hops but longer hop distances [53].

Nevertheless, it is not always possible to find such a route in static sensor networks topology. Therefore, an adaptive self-configuration topology mechanism is important for CR-WSNs for obtaining scalability, reducing energy consumption and achieving better network performance. An adaptive self-configuration topology mechanism performs better than static topology, even though it is a challenging issue to design and implement. This area has not received much research attention.

### 3.4. Fault Tolerance

CR-WSNs should have self-forming, self-configuration and self-healing properties. In other words, whenever some nodes or links fail, an alternative path that avoids the faulty node or link must be derived. In CR-WSNs, faults can occur for a variety of reasons, such as hardware or software malfunctioning, or natural calamities, e.g., fire, floods, earthquakes, volcanic eruptions, or tsunamis *etc.* A CR-WSN should always be prepared to deal with such situations. There are several types of faults, such as node fault, network fault, and sink fault, *etc.* Souza *et al.* [54] surveyed the well fault tolerance in WSNs. Hoblos *et al.* [55] modeled the fault tolerance or reliability *R_k_(t)* of a wireless sensor node using the Poisson distribution within the time interval (0,t) as follows:

(4) $$R_{k}(t)=\exp(-\lambda_{k}t)$$

where *λ_k_* is the failure rate of wireless sensor node *k* and *t* is the time period.

The fault tolerance is one of the challenging issues in CR-WSNs. The protocols designed for CR-WSNs should have a level of fault tolerance capability so that the overall function of the WSNs should not be interrupted.

### 3.5. Manufacturing Costs

Generally, CR-wireless sensors have been deployed in large numbers. Therefore, the cost should be significantly low. In contrast to conventional WSNs, which require less memory and computation capability, CR-WSNs require moderate memory and computational capabilities. To reduce the hardware cost, an algorithm that requires less computational power and memory should be developed. Designing such an algorithm is a challenging issue. Furthermore, CR wireless sensors should contain intelligent radio, application specific positioning systems (e.g., GPS), energy harvesting unit *etc.* which obviously increase the production cost.

### 3.6. Clustering

Logically grouping and organizing similar CR wireless sensors in their proximity has several advantages. Grouping sensor nodes into clusters has been pursued in WSNs to achieve the network scalability, reduce energy consumption and reduce the communication overhead. Several types of clustering exist, such as static, dynamic, single hop and multihop, homogeneous and heterogeneous. Very little work has been done in this area, which is also one of the research challenges.

### 3.7. Channel Selection

Because there is no dedicated channel to send data, sensors need to negotiate with the neighbors and select a channel for data communication in CR-WSNs. This is a very challenging issue, because there is no cooperation between the PUs and SUs. PUs may arrive on the channel any time. If the PU claims the channel, the SUs have to leave the channel immediately. Therefore, data channels should be selected intelligently considering the PU's behavior on the channel and using some AI algorithms.

### 3.8. Scalability

For some applications, CR wireless sensors should be deployed in huge numbers. Unlike conventional WS nodes, CR wireless sensors require cooperation among nodes for spectrum information sharing. This is very difficult to coordinate in a heterogeneous CR wireless sensor environment. Algorithms and protocols developed for CR-WSNs should be capable of solving these issues due to the growing size of the network.

### 3.9. Power Consumption

CR wireless sensors are power constraint devices with a limited energy source. In addition to the energy needed for spectrum sensing, channel negotiation, route discovery, transmission and reception of data packets, backoff, and data processing, CR wireless sensors also require energy for frequent spectrum handoff. A CR wireless sensor needs to sense the PUs' activities on the channel. Many applications require multiple antennas to monitor the PUs' activities, hence more energy is consumed.

Although there are several proposals for energy harvesting [56–58], these techniques have their own limitations. In some application scenarios, energy harvesting or the replenishment of power resources is not possible. Such limitations are well studied in [59–61].

In *ad hoc* CRNs, power consumption is an important design factor, but not the primary consideration. However, in CR-WSNs, it is one of the main performance metrics that directly affect the network lifetime.

### 3.10. Quality of Service (QoS)

In conventional WSNs, the QoS is generally characterized by four parameters: bandwidth, delay, jitter and reliability. To avoid hazardous consequences in critical applications, WSNs need to maintain an adequate level of QoS. QoS support is a challenging issue due to resource constraints, such as processing power, memory, and power sources in wireless sensor nodes. This is more challenging in CR-WSNs because in addition to the challenges in WSNs, it has one more challenge to protect the rights of PUs to access the incumbent spectra. PU's communication should be interference free with the SUs. This is more challenging because it is difficult to predict the PU's arrival on the channel. A miss-detection of the primary signal and a false alarm can cause additional challenges.

### 3.11. Security

Wireless sensors are normally deployed in an unattended environment, and are prone to security and privacy issues. CR wireless sensors can be attacked physically, and the data can be stolen. CR-WSNs are more vulnerable to security threats than the conventional WSNs, because there is no strict cooperation between PUs and SUs communication. The data collected by CR wireless sensors can be sniffed, destroyed or altered by unauthorized entities. In addition, attackers can interfere with PUs transmission or prevent the use of the channel by SUs through spectrum sensing data falsification (SSDF). CR-WSNs should have some acceptable level of security robustness against these potential threats and attacks. In addition to the security issues in conventional WSNs [53], additional security challenges are there for CR-WSNs. Some of the security issues in CR-WSNs can be as follows: (a) unacceptable interference to licensed users; (b) prohibiting the use of idle channels for SUs; (c) prohibiting the use of common control channels by virtually creating a bottleneck problem; (d) access to private data; (e) modification of data; and (f) injection of false data. Some possible attacks in CR-WSNs are discussed below.

#### 3.11.1. Physical Layer Attack

A jamming attack is one type of attack on a physical layer (PHY). In this type of attack, the adversary transmits radio signals on the wireless channels to interfere with CR wireless sensors normal operations. These adversaries may have powerful and sophisticated hardware and software. If the adversary blocks the entire network, it constitutes DoS attack at PHY. Another type of attack on PHY is a tampering attack. In this, the attacker may damage the CR wireless sensors, replace the entire nodes, or part of its hardware.

#### 3.11.2. MAC Layer Attack

In a MAC layer attack, the attackers violate the rules defined in the protocols and disturb the normal operation, e.g., sending undesirable packets repeatedly to disturb the normal operation and exhaust battery power, using channels selfishly, and show uncooperative behavior on the priority of a cooperative MAC-layer. This may lead to DoS attacks at the MAC layer.

#### 3.11.3. Routing Layer Attack

In a routing layer, the attackers alter the information on routing packets and misguide the packet forwarding sensors on the networks. The same things can happen to data packets as well. The following routing layer attacks are possible in CR-WSNs

(a) Wormhole attack: In a wormhole attack, an attacker builds bogus route information and tunnels the packet to another location. This creates routing loops and wastes energy.
(b) Sinkhole attack: In a sinkhole attack, the adversary provides false information to the wireless sensor nodes in the networks, such as it has the shortest route or efficient route *etc.*
(c) Sybil attack: In a Sybil attack, a single node may present multiple identities to the other nodes in a network. This may mislead the geographic routing protocols as the adversary appears to be in multiple locations at the same time.

These issues are entirely open research issues, and need to be explored further to meet its research challenges.

### 3.12. Sensing Techniques

**Figure 5.**
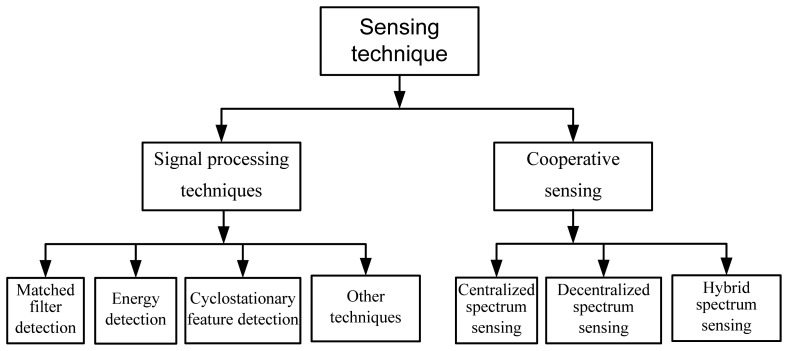
Classification of spectrum-sensing techniques.

One of the main objectives of imbedding a CR in a wireless sensor is to utilize the unused licensed spectrum opportunistically. Here, opportunistically means the SUs should protect the accessing right of the PUs whenever necessary. The interference of SUs to PU depends on the sensing accuracy of SUs. If SUs can sense the channels with high accuracy, interference with the PU decreases. Depending on the sensing technique, there is a tradeoff between the sensing delay and sensing accuracy. The technique that takes a long sensing time has more accuracy with the cost of delays and vice versa. Basically, there are two types of sensing techniques: (a) signal processing techniques; and (b) cooperative sensing techniques. As shown in Figure 5, signal processing sensing techniques for CR-WSNs can be divided further into matched filter detection, energy detection, cyclostationary feature detection and some other techniques. Similarly, cooperative sensing can be divided further into centralized spectrum sensing, decentralized spectrum sensing and hybrid spectrum-sensing techniques.

#### 3.12.1. Signal Processing Techniques

(a) Matched filter detection: The matched filter detection technique requires a demodulation of the PU's information signal at PHY and MAC layers, such as the modulation type and order, pulse shaping, packet format, operating frequency, bandwidth, *etc.* CR wireless sensors receive that information from the PU's pilots, preambles, synchronization words or spreading codes *etc.* The advantage of the matched filter method is that it takes a short time and requires fewer samples of the received signal. This decreases the received signal SNR and number of signal samples required. However, matched filter detection techniques consume considerable power and require high computational complexity and perfect knowledge of the target users.
(b) Energy detection: Energy detection detects the signal based on the sensed energy. This does not require prior knowledge of the PU's signals. This is a very popular technique because of its simplicity. The main disadvantage of this technique is its lower accuracy. Energy detection cannot discriminate the PUs' signal from the SUs' signal. This technique cannot be used to detect spread spectrum signals and has poor performance under a low SNR.
(c) Cyclostationary feature detection: In cyclostationary feature-detection techniques, modulated signals are coupled with sine wave carriers, pulse trains, repeated spreading, hopping sequences, or cyclic prefixes. The cyclostationary feature detection technique provides better performance, even in low SNR regions. This has good signal classification ability. However, it is more complex than energy detection and high speed sensing cannot be achieved. This cannot work if the target signal's characteristics are unknown.

#### 3.12.2. Cooperative Sensing

CR wireless sensors may encounter incorrect judgments because radio-wave propagation through the wireless channels has adverse factors, such as multi-path fading, shadowing, and building penetration. In addition, CR wireless sensors are hardware constraints and cannot sense multiple channels simultaneously. Therefore, CR wireless sensors cooperate and share their sensing information with each other to improve the sensing performance and accuracy. Three types of cooperative sensing exist: (a) centralized cooperative spectrum sensing; (b) decentralized cooperative spectrum sensing; and (c) hybrid cooperative spectrum sensing.

In centralized spectrum sensing techniques, each CR wireless sensor performs its own local spectrum sensing independently and makes a decision as to whether the PU's signal is present or absent on a particular channel. The CR wireless sensors then forward their decisions to a central cooperator, such as a cluster head, collector, or server. The central cooperator fuses the received decision of the CR wireless sensors and makes a final decision to infer the absence or presence of the PU. Whenever a CR wireless sensor wants to send data, it request channel information to the central cooperator.

In decentralized cooperation, the CR wireless sensors share intra-cluster information among clusters. CR wireless sensors in a cluster perform local spectrum sensing and inform the other CR wireless sensors within the clusters of their spectrum decision. There is no central cooperator; therefore it works in a decentralized manner. This scheme requires a periodic update on the spectrum information table, hence requires more storage and computation.

In hybrid cooperation, CR wireless sensors share information in a decentralized manner. However, the central cooperator may request a cluster head to send channel information whenever necessary. Although, cooperative sensing has advantages over non-cooperative sensing, it requires additional computation and resources, which is a challenge in hardware constraint CR wireless sensors.

## 4. Literature Review: Other Research on CWSN

As explained earlier, the CRWS network is still in its infancy. This area has received considerable research attention, and many researchers are working in different aspects of the CR-WSNs. This section discusses some of the influential research in the literature.

Vijaya *et al.* [6,62], Cavalcanti *et al.* [63], and Jia *et al.* [64] proposed prospective, survey, and key technologies on CR-WSNs. Bicen *et al.* [8] discussed the design challenges and principles for multimedia and delay-sensitive data transport in CR-WSNs in a range of environments. Liang *et al.* [10] proposed CR-WSNs, which can guarantee the QoS of both real-time traffic and best effort traffic. Feng *et al.* [65] suggested two resource allocation policies for supporting real-time constant-bit rate traffic in CR-WSNs.

Rashid *et al.* [66] evaluated the data link layer QoS performance of cognitive users, such as the average throughput and packet loss rate. In this model, the PUs' behavior is demonstrated as a two-state Markov Chain. The authors assumed that if the channel is not used by PUs at the beginning of a time frame, it remains unoccupied during the transmission of cognitive users. This is a conservative assumption because the PUs arrival is a random walk that can occur anytime.

Liang *et al.* [67] extended the reservation-based method proposed in [10,68]. The authors analyzed the transmission delay performance of CR-WSNs for supporting two types of real-time traffic, (a) bursty random traffic; and (b) Poisson traffic. However, they focused on the transmission delay of single cluster only.

### 4.1. Sensing Techniques

CR wireless sensors are energy constraints and opportunistic in nature. Hence, channel sensing techniques for the conventional WSNs or *ad hoc* CR networks may not be suitable. Several authors proposed different sensing schemes. This section reviews these schemes.

#### 4.1.1. Signal Processing Technique

Li *et al.* [69] proposed an algorithm that estimates the interference temperature using a generalization of the direction of arrival (DOA) algorithm by considering cooperative frequency spectrum sensing based on a spatial spectral estimation. Their simulation results show that this algorithm can acquire a 30% gain in the ratio of frequency spectrum utilization than the conventional method. Zahmati *et al.* [70] presented a hybrid sensing method that finds the optimal sensing period according to the characteristics of both PU and SUs. The proposed method varies its parameters adaptively to avoid unnecessary sensing tasks based on a continuous time Markov chain model. Zhang *et al.* [71] introduced the concept of a joint source and channel sensing (JSCS) for CR-WSNs. A specific slotted sensing and transmission scheme delivers the application source information to the access point energy efficiently. Hu *et al.* [72] proposed a new spectrum sensing scheme for CR-WSNs based on a spatially-decaying, time-incremental updating algorithm. This algorithm automatically assigns weights to channel information based on the distance between the source node and observing node. This algorithm is an extension of gossiping updates for an efficient spectrum sensing scheme that adopts the Flajolet-Martin aggregation to reduce the volume of data. Ma *et al.* [73] proposed spectrum sensing in OFDM based on energy detection in MIMO CR-WSNs. The OFDM-based MIMO CR-WSNs detect the primary user OFDM signal, where the CR receiver is equipped with a multiple antenna-based energy detector. They examined the soft combination of the observed energy values from different cognitive radio users and proved that square-law-combining is almost optimal in the low SNR region.

#### 4.1.2. Cooperative Sensing

A few cooperative sensing schemes are available in the literature. Some of them are discussed here. Thuc and Insoo [74] proposed a censor-based cooperative spectrum sensing scheme using fuzzy logic for CR-WSNs. They proposed a Takagi-Sugeno's fuzzy system to make a decision on the presence of the PU's signal based on the observed energy at each CR wireless sensor. In this scheme, the local spectrum sensing results aggregated at the fusion center after being censored to reduce the transmission energy and reporting time to make a final sensing decision. Wang *et al.* [75] also proposed a similar cooperative spectrum sensing scheme based on fuzzy logic for CR-WSNs. In this scheme, the CR wireless sensors use T-S fuzzy logic to make local decisions on the presence or absence of the PU's signal, and use a censoring method to allow only relatively reliable decisions sent to the fusion center.

A problem in cooperative sensing is that selfish wireless sensors have the option to choose cooperative spectrum sensing or local spectrum sensing. A selfish wireless sensor selects whatever it determines to be more profitable. How to achieve a desired decision outcome that maximizes spectrum utilization under the requirement of self-interest maximization and PU protection is discussed in reference [76]. The authors propose a revenue function that evaluates the gain and cost of a wireless sensor in choosing cooperative sensing and local sensing. The gain comes from data transmission and the cost comes from the delay and energy consumption. The interactive decision-making of wireless sensors is formulated as a noncooperative game, and the Nash equilibrium corresponds to a stable decision outcome in the sense that no wireless sensor is willing to unilaterally deviate from it.

Hareesh and Singh [77] proposed a hybrid cooperative detection scheme that associates the Eigen value-based spectrum sensing with an energy detector. This can be implemented for a range of signal detection applications without knowledge of the signal, channel and noise power. Maleki *et al.* [78] proposed a cooperative spectrum sensing scheme for CR-WSNs. In this scheme, CR wireless sensors detect the channels using an energy detection technique. The sensing results of each CR wireless sensor are collected at the fusion center, which makes a global decision on the presence of the PU on the channel using a fusion rule. This scheme employs hard decision based spectrum sensing, in which the sensor nodes send one bit per decision, unlike a soft decision scheme that sends multiple bits per decision. They also proposed a sleeping and censoring scheme for energy efficiency.

### 4.2. Energy Efficiency

As the importance of energy conservation in CR-WSNs has been discussed, several schemes for energy efficiency have been proposed. They use diverse techniques, albeit the goal of these schemes is to conserve energy. Some of the techniques are discussed here.

#### 4.2.1. Energy Efficiency in Sensing

Energy efficient spectrum sensing technique is a basic requirement for the CR-WSNs. Izumi *et al.* [79] proposed a low-power multi resolution spectrum sensing (MRSS) architecture for CR-WSNs. Unlike the conventional MRSS scheme, which consumes considerable power and is comprised mainly of analog circuits in which a sensing bandwidth and sensing sensitivity are altered by an analog variable filter, the scheme proposed in this paper carries out signal processing in a digital domain and can detect occupied frequency bands at multiple resolutions and with low power consumption.

#### 4.2.2. Clustering for Energy Efficiency

As described in Section 3, the routes with more hops and shorter hop distances can be more power efficient than those with fewer hops and longer hop distances. Therefore, clustering is one of the solutions for power conservation in CR-WSNs. Clustering schemes for WSNs cannot be used directly in CR-WSNs because to form a cluster, the sensors need to communicate and decide the cluster head in a different manner than the conventional WSNs. Zhang *et al.* [80,81] proposed distributed spectrum-aware clustering schemes considering the energy efficiency for CR-WSNs. The authors modeled the power consumption and derived the optimal number of clusters as follows:

(5) $$K_{\text{opt}}=\left\lfloor \frac{N}{r_{\max}\sqrt{3\rho }}+0.5\right\rfloor$$

where *K_opt_* is the optimal number of clusters, *N* is the number of CR wireless sensor nodes, *r_max_* is the maximal transmission range of CR wireless sensor nodes, and *ρ* is the CR wireless sensor nodes density. These schemes have low complexity and rapid convergence under dynamic PU activity.

#### 4.2.3. Energy Efficient Modulation Technique

Gao *et al.* [82] proposed an energy-efficient and adaptive modulation technique for CW-WSNs to achieve power efficiency. The authors proposed a subcarrier detection mechanism, where the user determines its optimal subcarrier, and minimize power consumption at each node by adjusting the constellation size using a modulation technique.

#### 4.2.4. Energy Efficient Packet Size Optimization

Oto *et al.* [83] formulated energy-efficient optimal packet size analytically. They also discussed the energy-efficient packet size optimization problem for CR-WSNs considering the acceptable interference level for PU and the maximum allowed distortion level between the event signal and its estimation at a sink node. A sequential quadratic programming method is used to determine the energy-efficient optimal packet.

#### 4.2.5. Energy Harvesting

RF energy harvesting enables the wireless sensor node to operate with a potentially perpetual lifetime. Park *et al.* [84] examined an optimal mode selection policy for CR-WSNs powered by RF energy harvesting. Assuming that the wireless sensor node harvests the RF energy received from the primary network, wireless sensor nodes can take advantage of the spectrum occupancy of the primary network for both idle and occupied states.

Nevertheless, CR wireless sensors are energy constraints, and the network lifetime basically depends on energy. Most of the energy efficient schemes focus on energy efficiency in a specific operation, such as channel sensing or data transmission, *etc.* To increase the energy efficiency, energy conservation on several aspects of network operation should be considered, including channel sensing scheme, clustering and topology management algorithm, routing algorithm, MAC protocols, channel selection and data reception, *etc.*

### 4.3. Spectrum Management and Channel Selection/Assignment

**Figure 6.**
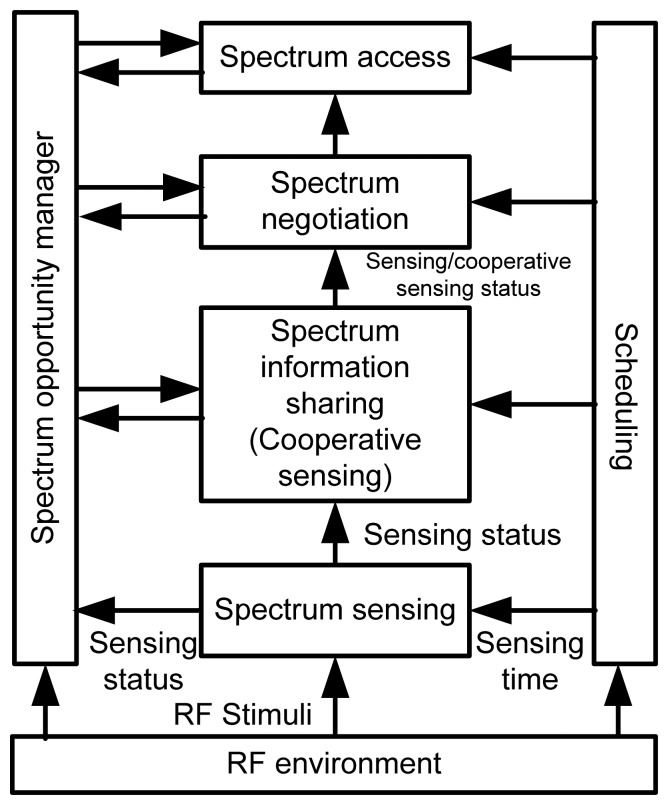
Logical framework of spectrum management.

Figure 6 shows the logical framework of spectrum management. With the distinct QoS requirements of different applications, the immense need of a spectrum allocation scheme can fulfill the flexible bandwidth requirements.

Byun *et al.* [85] proposed a centralized spectrum allocation scheme for CR-WSNs. Wireless sensor node request a spectrum resource to the coordinator. The dedicated coordinator allocates the spectrum resources to the sensors. The central coordinator is responsible for fairness, efficient spectrum utilization and spectrum handoff. Modified game theory is used for spectrum allocation.

A channel allocation scheme for CR-WSNs is proposed in [86]. The authors proposed two tier sensor networks, the lower tier has the WSNs and the upper tier has the CR nodes responsible for monitoring the environment. The CR nodes in the upper tier compete for spectrum to relay their messages to the sink node. The authors incorporated two constrained Markov decision processes: one for accurately detecting an event while satisfying the delay, PFA and congestion constraints by adjusting the detection criterion; and the other for allocating the best available spectrum to the SU on a priority basis.

Wu *et al.* [87] proposes a scheme for data channel assignment, where each wireless sensor node selects a channel considering the channel utilization and network connectivity of the PU. The channel information is obtained from a gateway node or a sensor node closer to the gateway than the current node. The data can be delivered using multiple channels.

In this scheme, a gateway node broadcasts a channel assignment request message (CAR) using the control channel. The message consists of a gateway address, channel number and available bandwidth. Upon the reception of a CAR message, each node calculates the available channel list using the information contained in the message. If a node has a reliable direct link to the gateway, the node adds all candidate channels announced by the gateway. Otherwise, the node adds a channel to its available channel list only when there is a reliable link to the upstream node, which uses the same channel. When a wireless sensor node is not a reliable neighbor of the gateway, the node assigns itself a channel upon the reception of a channel information notification (CIN) message. Once the working channel at a wireless sensor node is determined, the wireless sensor node broadcasts a CIN message using the control channel and then switches to the data channel.

Li *et al.* [88] examined the channel assignment problem in a cluster-based multi-channel CR-WSNs considering energy consumption. An R-coefficient was proposed to estimate the predicted residual energy using sensor information (current residual energy and expected energy consumption) and the behavior of the PU on the channel. Based on the R-coefficient, three channel assignment approaches are provided: random pairing, greedy channel search and optimization-based channel assignment. However, this scheme does not guarantee fairness among sensors.

Han *et al.* [11] proposed an energy-efficient channel management scheme for CR-WSNs. In this scheme, CR wireless sensor node adaptively selects its operation mode among channel sensing, channel switching and data transmission/reception, according to the channel sensing outcome. The authors proposed the scheme based on the partially observable Markov decision process framework, considering that the sensing outcome can be erroneous due to noise uncertainty.

### 4.4. Channel Access

The MAC protocols in CR-WSNs are different than that in conventional WSNs and CR *ad hoc* networks in several ways. The conventional MAC protocol for WSNs depend basically on the physical layer (PHY). However, only carrier sensing is not sufficient in CR-WSNs because the node needs to have complete knowledge about the spectrum availability. Conventional WSNs simply retransmit packets after a collision, but in CR-WSNs, the node needs to determine if the collision is with the PU or SU. If the collision is due to the PU, SU has to leave the channel immediately. Only small amount of work has been done in this field.

Motamedi and Bahai [89] formulated an optimization model for energy-efficient spectrum access to minimize the energy per bit for each single user. However, this network model considers and ignores the PU behavior. The channel selection decision is made individually without considering the collisions to other cognitive users and the energy consumption based on the entire network. Hu *et al.* [90] presented a dynamic spectrum access strategy based on the real-time usability considering the spectrum idle condition and communication capability. An energy-saving algorithm for the spectrum utilization was proposed. Shah and Akan [91] reported the performance of the CSMA-based MAC protocol with CCC for CR-WSNs. In this protocol, the two performance metrics were derived based on the fact that the SUs can exploit the cognitive radio to simultaneously access distinct traffic channels in the common interference region. Gao *et al.* [92] extended their previous work [82], where they proposed an adaptive modulation technique for CW-WSNs, by allowing each user to choose multiple subcarriers for data transmission. Considering that new users in the network can choose the same subcarriers in the same time slot independently, and co-channel interference can occur, this scheme allows multiple new users to share the same subcarriers provided their respective SINR is acceptable.

### 4.5. Common Control Channel in CR-WSNs

In any type of CRN, a common control channel is necessary for spectrum sensing information sharing, transmitter–receiver handshake, neighbor discovery, channel access negotiation, clustering, topology change, routing information updates, emergency message broadcasting *etc.* Moreover, a common channel is used to send control messages to the neighbors to inform the state of the operation, and the destination for facilitating the continuous operation of the SUs without interruption.

The common control channels can be classified into two types:

(a) Common control channel: A channel temporally and opportunistically used by SUs. This is common between at least two SUs. This is not dedicated only for the SUs' control message exchange. Therefore, PUs can use it anytime.
(b) Dedicated common control channel: A dedicated channel used mainly for control packet exchange. This channel is common among all the SUs in the networks. This channel is considered as being free of PU interference and always available for SUs.

A dedicated common control channel is essential for reliable communication in CRNs. Several methods can be used to obtain a common control channel: (a) Acquire or rent a dedicated band; (b) select a channel from the incumbent licensed band using a hopping sequence; (c) use an underlay approach, such as the UWB radio technology; and (d) use the ISM band. A common control channel can be saturated [93] under some network conditions. Therefore, non-dedicated common control channel based MAC protocols [46] are suggested.

### 4.6. Routing in CR-WSNs

Routing protocols are required to discover and maintain the routes in CR-WSNs. Routing in CR-WSNs is quite challenging due to the inherent characteristics that distinguish CR-WSNs from other wireless networks, such as *ad hoc* CRNs and WSNs. In CR-WSNs, in addition to the number of hopes and energy consumption, the CR wireless sensor nodes need to consider the number of channels available for SUs on a particular route. Some of the existing works are described here.

Quang *et al.* [94] proposed a routing algorithm for CR-WSNs that selects an optimal path to forward packets to the sink based on stochastic characteristics of the primary channels. The algorithm requires dedicated fixed cluster head equipped with an external energy source, which makes this protocol difficult deploy under certain conditions.

Oey *et al.* [95] proposed a routing protocol that is similar to the AODV protocol. In this protocol, the authors considered 15 unlicensed channels and one licensed channel for data transmission and one unlicensed channel for the common control channel of CR-WSN. Their assumption was quite conservative in that they assumed that 70% of the unlicensed channels are available. In addition, they just considered one licensed channel with the remainder being unlicensed channels. The unlicensed channel is used for CCC, which cannot be available all the time. Some other routing protocols have also been proposed [87,96–98].

### 4.7. Security and Trust Issues

Very little work has been done in security and trust issues in CR-WSN. Sen [99] presented a comprehensive discussion on the security and privacy issues in CWSNs by identifying a range of security threats in these networks and various defense mechanisms to counter these vulnerabilities. The author categorized the various types of attacks on CWSNs under different classes based on their natures and targets, and appropriate security mechanisms corresponding to each attack class are also discussed.

Al-Qasrawi *et al.* [100] discussed the security challenges of CWSN and proposed a new cognitive wireless sensor system paradigm with many techniques, which proved their efficiency separately to face the key challenges and threats on CWSN, particularly the security aspects. Lang *et al.* [101] has also performed some preliminary work in this area.

## 5. Coexistence with IEEE 802.22 (WRAN) and Other CR Networks

Research on the use of various CR devices in ISM and incumbent bands has been performed, but more research on the coexistence of CR devices operating in the same location will be necessary. Widely deployed CR wireless sensors use lower transmission power than other wireless network devices. Therefore, the coexistence issue between themselves and other non-CR-WSNs should be considered.

In addition to interference, there could be opportunities to utilize the spectrum information with the cooperation of IEEE 802.22 CPEs. Although there are no reports on the possibilities of the coexistence of CR-WSNs with IEEE 802.22 RAN, it may be possible to obtain information from the CPEs and/or WRAN BS and use the spectrum information. The CPE can work as a coordinator or gateway between the CR-WSNs and WRANs.

## 6. Research Trends and Open Research Issues

**Figure 7.**
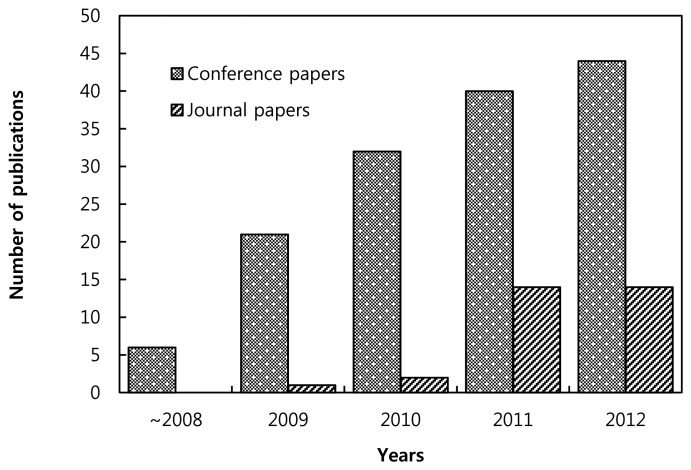
Number of research papers related to WSCRNs published in IEEE journals.

Although a number of papers have been published in this area, still many research issues remain to be addressed. Figure 7 shows the number of research papers published over the last few years. Less than 15 papers were published in IEEE journals in 2011 and 2012.

No clear standard exists and there have been several unclear proposals. Many areas need to be explored, such as low computational and energy efficient spectrum sensing, spectrum management, clustering, energy consumption, spectrum handoff, channel allocation, channel access, geo-location information sharing, self-topology generation, cross-layer optimization of protocol stacks, *etc.* In addition, many issues remain to be resolved, such as coexistence with other CR systems, legal issues to access incumbent channels, limit of interference with PUs, transmission power control *etc.*

## 7. Conclusions

A CR wireless sensor network is a type of wireless sensor network that comprises spatially-distributed autonomous CR equipped wireless sensors to monitor the physical or environmental conditions cooperatively. This paper discusses the evolution of CR-WSNs, opportunities, technical issues, research trends and challenges. Some of the recent research results in CR-WSNs were surveyed. CR wireless sensor networks are still in their infancy. Several areas remain to be explored and improved. For the success of CR-WSNs, massive research is required in several aspects. Substantial developments in hardware, software and algorithms are needed to make smart CR wireless sensors. The following are the potential challenges for the success of CR-WSNs

- Development of a wireless sensor with the required cognitive capabilities,
- Development of extremely low power consumable CR wireless sensor with energy harvesting facilities,
- Capability of operating at high volumetric densities,
- Producing low cost CR wireless sensors,
- Development of autonomous and unattended operable algorithms and protocols,
- Highly intelligent and adaptive to the environment
- Should be robust on security for attacks and should work in an untrustworthy environment,
- Development of globally operable CR wireless sensor *etc.*

This paper is expected to provide research directions in the CRWS network area.

## Figures and Tables

**Table 1.** Prospective capabilities of a wireless sensor with CR.

**Function**	**Action**
Cognitive capabilities	
Spectrum sensing	Detect unused spaces (white spaces) by the incumbents in the spectrum bands.
Spectrum sharing	Use the unused white spaces of incumbents and share the white space information with cognitive users.
Prediction	Predict the arrival of incumbents on the channel.
Fairness	Distribution of spectrum utilization opportunities fairly among cognitive users.
Routing	Route the packet to the destination efficiently considering the network life span, load balancing, shortest route and delay in multi-hop CR-WSNs.
Reconfiguration capability	Reconfigure and adjust according to the environment outcomes.
Environment sensing	Sensing the environmental factors as in conventional wireless sensors *.
Trust and security	Building a trustable environment and secure networks.
Power control	Control transmission power considering the legal boundaries and requirements.

* application specific.

**Table 2.** Frequency bands available for ISM applications, as defined by ITU-R.

**Range**	**Center Frequency**	**Bandwidth**
6.765–6.795 MHz	6.78 MHz	30 kHz
13.553–13.567 MHz	13.56 MHz	14 kHz
26.957–27.283 MHz	27.12 MHz	326 kHz
40.66–40.7 MHz	40.68 MHz	40 kHz
433.05–434.79 MHz	433.92 MHz	1.84 MHz
902–928 MHz	915 MHz	26 MHz
2.4–2.5 GHz	2.45 GHz	100 MHz
5.725–5.875 GHz	5.8 GHz	150 MHz
24–24.25 GHz	24.125 GHz	250 MHz
61–61.5 GHz	61.25 GHz	500 MHz
122–123 GHz	122.5 GHz	1 GHz
244–246 GHz	245 GHz	2 GHz

**Table 3.** Comparison of *ad hoc* CRNs, WSNs and CR-WSNs.

**Factor**	***Ad Hoc*CRNs**	**WSNs**	**CR-WSNs**
Wireless medium	Licensed spectrum bands (Data channels) Licensed or ISM band (control channel)	ISM bands	Licensed spectrum bands (Data channels) Licensed or ISM band (control channel)
Traffic	Random	One to many, many to one, many to many	One to many, many to one, many to many
Hardware constraints	Intelligent *ad hoc* mobile devices with cognition capability	Small, low processing capacity, low memory capacity	Intelligent, cognition capabilities, small, moderate processing capacity, moderate memory capacity
Availability	Under development	Readily available	Not readily available (under conceptual phase)
Bandwidth deficient	Yes	Sometimes	Yes
Identification	Unique ID by its MAC address	Not unique	Not unique
Standards	Not yet defined	ZigBee, IEEE 802.15.4, ISA100, IEEE 1451	Not yet defined
Fault tolerance	Less critical points of failure	High fault tolerance required	High fault tolerance required
Communication Range	Long	Short	Short (intelligently controllable)
Communication	Broadcast	Point-to-Point	Point-to-Point
Failure rate	Low	High	Moderate (*expected)
Population of nodes	Sparsely populated	Densely populated	Densely populated
Interaction	Close to humans e.g. laptops, PDAs, mobile radio terminals, *etc.*	Focus on interaction with the environment	Focus on interaction with the environment
Topology changes	Frequent	Less frequent	Less frequent
Seamless operation	Depends on the PUs	Not concerned with PUs	Depends on the PUs
Suitable for	Where ISM band is overcrowded	Where ISM band is not crowded	Where ISM band is overcrowded
Whitespace utilization concern	Yes	No	Yes
Data centric	Generally address-centric networking	Generally data-centric	Generally data-centric
Application specific	Generally not	Yes	Yes
Self-organization	Cognitive decision support system	Yes, but no cognitive decision support system	Cognitive decision support system
Multi-hop communication	Often	Often	Often
Energy conservation	Concern	Highly concern	Highly concern
Trust/Security	Usually, no central coordinator	One administrative control	One administrative control
Mobility	Often (MANET)	Less mobile or stationary	Less mobile or stationary
Routing	All-to-all	Broadcast/Echo from/to sink	Broadcast/Echo from/to sink
Multichannel	Required	Possible	Required
CCC requirement	Mostly Required (except some exceptions) [17]	Not really	Mostly Required (except some exceptions)
In-network processing	Supposed to deliver bits from one end to the other	Expected to provide information on the other end, but not necessarily original bits	Expected to provide information on the other end, but not necessarily original bits
Scalability	Not many (10s to 100s of nodes)	Very large (10s to 1,000s)	Very large (10s to 1000s)
QoS interpretation	Receipt rate, Dissemination, Speed, Spectrum utilization, Interference to PUs	Energy consumption, Redundancy Efficiency, Latency, Scalability, Robustness - Event detection/reporting probability - Event classification error, detection delay - Probability of missing a periodic report - Approximation accuracy - Tracking accuracy	Energy consumption Redundancy Efficiency, Latency, Scalability, Robustness Throughput/Delay - Event detection/reporting probability - Event classification error, detection delay - Probability of missing a periodic report - Approximation accuracy - Tracking accuracy - Spectrum utilization - Interference to PUs
Research direction	Many areas are still to explore Currently focus of research is predominantly directed towards – Game theoretic approaches for spectrum utilization – Predictions for the PUs arrival – Energy efficient routing and MAC protocols – Development of middleware architectures – Distributed aggregation applications – Design of cross-layer algorithms for improved power efficiency	Although, there is always room for improvement, most of the areas are explored and now research focus on – QoS, – reliability, – performance enhancement – trust and security – *etc*.	Research is still in infancy Almost all area are still to explore Currently focus of research is predominantly directed towards – Game theoretic approaches for spectrum utilization – Predictions for the PUs arrival – Energy efficient routing and MAC protocols – Development of middleware architectures – Distributed aggregation applications – Design of cross-layer algorithms for improved power efficiency
